# Progress in Ophthalmology

**Published:** 1899-02-04

**Authors:** 


### Progress in Ophthalmology.

**Protargol in Ophthalmia Neonatorum and other Conjunctival Diseases.**—F. E. Chenery[1] of Boston, speaks well of protargol (a fine, yellowish powder, consisting of silver, combined with a protein substance) instead of nitrate of silver in the treatment of conjunctival diseases, and especially in cases of ophthalmia neonatorum. He says: "It does not stain the skin, and causes scarcely any irritation, so that a two per cent. to four per cent. solution can be used without cocain." He employed it in twenty-five cases of ophthalmia neonatorum, in ten of which one eye was treated with protargol and the other with nitrate of silver. The small amount of irritation in the eye treated with protargol was very noticeable, and there was less tendency to the formation of a fibrinous coagulate. As a prophylactic it was also most efficacious.

**New Method of Applying Nitrate of Silver in Conjunctivitis.**—Dr. Norburne Jenkins[2] proposes this method, which he says is much better than the aqueous solution of silver nitrate of Crede. He says probably the least injurious and most efficacious remedy in the treatment of all forms of purulent diseases of the conjunctiva is an emulsion the formula and directions for which are here given: ℞ Argent. nitratis gr. 5-10; acaciæ dr. 1 or qs.; aq. destill. fl oz ½; petrol liquidi fl. oz. ½ ℳ. Make an emulsion and put into a wide-mouthed bottle; shake well before using. When properly prepared the emulsion has the consistency of thick cream, and is milky-white in colour.

In the initial stage of blenorrhœa the customary treatment is to be followed—viz., iced compresses, Leiter's tubes, leeches; frequent cleansing with a mild solution of corrosive sublimate or permanganate of potassium. When suppuration is established the emulsion may be used in the following manner: The lids are everted and the eye is thoroughly cleansed with a 1-5,000 solution of corrosive sublimate. While the lids are still everted, one or two minims of the emulsion are dropped into the eye by an assistant. The surgeon then manipulates the everted lids for thirty seconds in such a manner that the emulsion may penetrate to the utmost recesses of the conjunctival folds. In this manipulation consists to a great extent the proper application and efficacy of the remedy. Another method is the application with a camel-hair pencil introduced under the everted lid into the fornix conjunctivæ. This he considers is a hazardous procedure when practised by any but the deftest fingers. Extreme care is to be observed that the conjunctiva or cornea be not wounded, as such injuries are shortly followed by exaggerated foci of infection.

**Formalin in Blepharitis.**—Dr. H. Moulton[3] recommends formalin in blepharitis as the result of his treatment of some obstinate cases during the last year. A solution varying from 2-10ths per cent. to 1 per cent. he thinks is best. Beginning with the lower strengths he gradually works up. To apply it he wraps a small piece of cotton wool round the point of a toothpick in the form of a mop, kept small so that it does not take up enough solution to run in on the conjunctiva. The solution must be frequently renewed or prepared fresh on each occasion to ensure uniformity of strength. The mop dipped in the solution is rubbed gently along the margin of the lid (which has been drawn slightly away from the eye) among the lashes till all the scales and crusts have been removed, and until the surface of any little pustule which may be present has been rubbed off. The site of the disease is then left clean and smooth. The mop is renewed once or twice during the operation. A little bland oil may be applied afterwards, or the formalin may be used in the oil. The applications are made daily if possible by the surgeon himself. Several of Dr. Moulton's cases had been treated by himself by other means with little or no benefit, but improved rapidly under the formalin treatment.

**Treatment of Corneal Ulcers.**—Mr. Percy Dunn[4] speaks very warmly of chinesol and eserine in the treatment of corneal ulcers. He says of the former, that one of its chief advantages is the potency of its germicidal action—a fact which has been incontestably proved by bacteriological investigation; in addition it is freely soluble in water, and thus handy to use; moreover it is non-caustic, and does not injure the skin of the hands, does not coagulate albumen, and is non-hygroscopic. Mixed with 1 in 20 boric acid it makes an admirable antiseptic ointment—1 in 2,000 seems to be the strength of the lotion he employs. As to the use of eserine, he says, the great point is to remember to use it in a weak solution (gr. ss.-ʒ). Furthermore, as to the form of ulcer, in which it is most useful he says all sloughing, infective, and vascular ulcers are best treated by eserine, as well, of course, as those situated at the corneal margin, and which threaten to perforate, and that this may be taken as a broad rule for guidance.

**Accommodation Theory of Helmholtz.** — At the Ophthalmological Society's meeting in November, Mr. Priestly Smith[5] read a paper on the Accommodation Theory of Helmholtz, as opposed to that of Tscherning, and suggested an explanation of their discrepancy. He said that Tscherning's theory was sharply opposed to the generally accepted explanation, but was based on very accurate observations. The question was whether the accommodation change in the lens depended on slackening of the zonula as stated by Helmholtz, or on its tightening as stated by Tscherning. The anatomical arrangement of the parts concerned, and nearly all clinical and experimental facts, showed that during strong accommodation the lens was tremulous and displaced downwards by gravity, pointing to the correctness of the slackening theory as held by Helmholtz. Tscherning demonstrated that during accommodation the anterior surface of the lens assumed a conical form, which he said could only take place when the zonula was tightened. Mr. Priestly Smith then demonstrated, by means of a steel hoop strengthened in various ways and bent to the shape of a lens during accommodation, that by slackening the pull on the equator of the lens the anterior surface of the lens assumed this conical form demonstrated by Tscherning. It would be easy to theorise as to the resistance proper to the crystalline lens and to show reason for supposing that the arrangement of the fibres was such as to render the resistance progressively greater towards the equator, but Mr. Priestly Smith said he would not go beyond demonstrable facts. What he desired to show was that the changes which Tscherning had demonstrated with such admirable skill and ingenuity were not incompatible with the theory of Helmholtz.

**The Prognostic Importance of the Appearance of Eye Lesions in Insterstitial Nephritis.**—A study of the statistics of this subject is not without interest. Two years ago Belt[6] was led by his search through the literature of the subject into feeling that a more hopeful prognosis might be given in cases of Bright's disease if strict adherence to the counsel and therapy of the physician was given. With this preconceived idea in view Belt brought together the data of 409 cases furnished by most of the prominent oculists of the United States. His hopes, however, were doomed to disappointment, for when the accumulated facts had been statistically arranged it was learned, that of 409 individuals the subject of albuminuric retinitis, due largely to living at the pace that kills, 155 were cases in private practice, of which number 62 per cent. died within one year, 85 per cent. in two years, and 15 per cent. lived more than two years. Of the 77 hospital cases 85 per cent. died within one year, 93 per cent. in two years, and 7 per cent. lived more than two years. Of the remaining 187 cases 65 per cent. died within one year, 93 per cent. within two years, and 7 per cent. lived more than two years. If these facts be properly collected it is plainly shown that the large majority of the victims of albuminuric retinitis die within the first year of the appearance of the eye lesion, fully 90 per cent. die within the expiration of two years from that time, while a scant 10 per cent. drag out a weary existence stretching over ten years sometimes, and even to seventeen years, as in the case reported by Webster. The foregoing then establish a scientific truth of twofold value. First, in that it enables the practitioner to prognose intelligently in a given case. Second, by reason of such authoritative prognosis, the sufferer, who is usually of the well-to-do class, may be acquainted with the suddenness with which his malady may terminate—permitting him to so arrange his worldly affairs as to set aside all necessity for the litigation that follows on the death of most of those who are unfortunate or improvident enough to die intestate.

[1] Brit. Med. Jour., Oct. 8, 1898. [2] Med. Rec., Oct. 29, 1898. [3] New York Med. Jour., Oct. 1, 1898. [4] Lancet, Oct. 22, 1898. [5] Brit Med. Jour., Nov. 19, 1898. [6] Internat. Med. Mag., June, 1898.

